# Measuring experience of and satisfaction with newborn care: a scoping review of tools and measures

**DOI:** 10.1136/bmjgh-2022-011104

**Published:** 2023-05-09

**Authors:** Nicole Minckas, Rashmi Kharel, Marcella Ryan-Coker, Ornella Lincetto, Özge Tunçalp, Emma Sacks, Moise Muzigaba, Anayda Portela

**Affiliations:** 1 Institute for Global Health, University College London, London, UK; 2 Department of Maternal, Newborn, Child and Adolescent Health and Ageing, World Health Organization, Geneva, Switzerland; 3 Deanery of Clinical Sciences, University of Edinburgh, Edinburgh, UK; 4 Department of Sexual and Reproductive Health and Research, World Health Organization, Geneva, Switzerland; 5 Department of International Health, Johns Hopkins University Bloomberg School of Public Health, Baltimore, Maryland, USA

**Keywords:** review, child health, health services research, health systems evaluation

## Abstract

**Background:**

Standardised measures on experience of care are essential to understanding the care women and newborns receive and to designing appropriate interventions and responses. This review builds on ongoing work in the realm of maternity care and complements it by reviewing existing tools and measures to assess experience of and satisfaction with the care of the newborn.

**Methods:**

We conducted a scoping review of published literature to identify measures and tools of experience (physiological or indirect) and satisfaction with newborn care. We systematically searched five bibliographic databases from 1 January 2010 through 1 December 2022 and contacted professional networks. Using a predefined evidence template, we extracted data on the studies and the tools’ characteristics. We mapped the tools and measures against the WHO quality of care frameworks to identify the most frequent measured domains of care and to highlight existing gaps.

**Result:**

We identified 18 292 records of which 72 were eligible. An innovative finding of this review is the inclusion of newborn perspectives through behavioural responses, physiological signals, pain profiles as well as other non-verbal cues as markers of newborn experience. Domains related to parental participation and decision-making, ensuring continuity of care and receiving coordinated care, were the most measured across the included tools.

**Conclusion:**

Comprehensive and validated instruments measuring all aspects of care are needed. Developing a robust theoretical ground will be fundamental to the design and utilisation of standardised tools and measures.

**Protocol registration details:**

This review was registered and published on protocol.io (dx.doi.org/10.17504/protocols.io.bvk7n4zn).

**WHAT IS ALREADY KNOWN ON THIS TOPIC**

- Positive experience of care is an essential aspect of quality of care and a key driver of health outcomes and future care-seeking behaviours.
- Many efforts have been undertaken towards improving women's experience of care and, in particular, to ensuring respectful person-centred care during childbirth. However, there is little evidence on what constitutes experience of newborn care and how to measure it.
- Measuring newborn experience of care is inherently faced with conceptual and pragmatic challenges, as it becomes necessary to identify who advocates on their behalf and recognise the link between theirexperience and that of the newborn.

**WHAT THIS STUDY ADDS**

- We included 72 articles from 34 countries across all regions, showing substantial variations on how experience and satisfaction were conceptualised and operationalised.
- Newborns’ non-verbal and physiological cues can be measured as an essential component of experience of newborn care.
- The experience of care of newborns can be interlinked with that of their mothers and/or carers’. Both should be considered to arrive to a broad and comprehensive understanding of experience of newborn care.

**HOW THIS STUDY MIGHT AFFECT RESEARCH, PRACTICE OR POLICY**

- Developing a robust theoretical ground will be fundamental to the design and selection of standardised tools and measures.
- Standardised measures on experience of care are essential to understanding the care women and newborns receive and to designing appropriate interventions and responses that inform policy and programme implementation.
- It is necessary to understand, measure and address the experience of care as a key driver of adverse health outcomes and a component of the right to health, equity and dignity.

## Introduction

In recent years, attention has been directed towards improving the quality of care for women and newborns during childbirth and the immediate postnatal period, with a focus on ensuring respectful person-centred care. The WHO has established eight standards for improving quality of maternal and newborn care in facilities to ensure a positive experience, which include three standards related to the experience of care: communication and participation (standard 4), rights and dignity (standard 5) and emotional support (standard 6).^1^ Although earlier studies have examined the physiological impact of the separation of mothers and newborns after birth, the importance of the overall experience of newborn care has only recently been recognised as a critical component of quality care.^2–5^

Despite the clear overlap of the type of treatment received by women and newborns, a literature review on mistreatment of newborns proposed that the core domains differ.^6^ For example, the typologies developed for women during childbirth tend to focus on events of direct abuse, whereas early evidence about disrespectful care of newborns suggests that mistreatment may be primarily around issues of neglect, separation from the mother/family, inappropriate feeding practices and absence of gentle, compassionate care or supportive environment.^1 7^ This emphasises the need to understand and measure newborns’ experience of care concurrently with that of the mother.

A recent review by Larson and colleagues^8^ compiled all available tools to measure facility-based experience of care for pregnant women and newborns.^8^ This review identified several undermeasured areas including newborn care. Thus, we considered it important to conduct an additional review on newborn experience of care for several reasons. First, to ensure that newborn care is adequately captured in the search, as certain facets of newborn experience were likely not captured in the previous review that focused on maternity care. Also, to explore the additional layer of complexity when it comes to defining ‘experience’ of newborn care and identifying who should advocate (health workers, parents or carers) on behalf of newborns by recognising the link between their own experience and that of the newborn. Finally, to assess the need to consider measures that capture non-verbal or physiological cues as a complementary strategy to capture aspects of experience of care, given the newborns’ inability to express his/herself verbally.

This review builds on ongoing work in the realm of maternity care and complements it by reviewing existing tools and measures to assess experience of and satisfaction with newborn care during the postnatal period.

## Methods

### Definition of terms

This scoping review covers two main concepts within the realm of quality of care: experience and satisfaction of newborn care. The study adopts the definitions of person-centred quality measures provided by Larson *et al*^9^ to frame the relationship between the two concepts. Experience of care is defined as a process indicator reflecting the interpersonal aspects of the quality of care provided.^9^ However, as mentioned, measuring the experience of newborns is a challenge due to their inability to verbally express their experiences. To address this, we defined the concept of *experience of newborn care* with two separate components: physiological experience and indirect experience. Physiological experience is defined as the biological measures that can be used to interpret the experience of newborns such as posture, movements, feeding, heart rate and respiratory rate, cortisol levels, sleep or cry patterns. Indirect experience, on the other hand, is defined as the experience of the mother, parents, carers and/or families with the care the newborn receives such as effective communication, respect and dignity, emotional support or being informed about the procedures performed on the newborn.^10^ Although we acknowledge that newborns can have direct emotional (non-physiological) experiences, it is challenging to measure it beyond the physiological cues or via the family’s experiences. The second concept explored is the overall *satisfaction of mother/parents/carers and families* with the care the newborn receives. Satisfaction is seen as an outcome measure influenced by experience of care, but notably also influenced by expectations and cultural norms.^9^

### Search strategy and selection criteria

We included articles reporting on tools (quantitative or qualitative), or measures related to experience of or satisfaction with newborn care. We included peer-reviewed articles or grey literature published on or after 1 January 2010 to ensure that the most current and widely used measures and methods were captured. We only considered original research, excluding editorials, comments or newspaper articles. Our eligible study population included newborns born either in facilities or at home and their parents/carers who sought or received care during the postnatal period (from birth up to 6 weeks after birth).^11^ We did not impose any restrictions on income level, geography or language. Reviews were excluded, but their reference lists were hand searched for potentially relevant studies.

The scoping review was conducted in accordance with the Preferred Reporting Items for Systematic Reviews and Meta-Analyses extension for Scoping Reviews guidelines (online supplemental appendix 1).^12^ We conducted a broad search in five databases (PubMed, Embase, CINAHL, Web of Science and Proquest) using a combination of Medical Subject Heading (MeSH) terms related to infant care including ‘Infant Health’, ‘Postnatal Care’, ‘Maternal-Child Health Services’ but without specifying particular domains that we were expecting to emerge from the evidence. Search terms were developed through consensus (NM, AP, ES, MM). The complete search terms used in PubMed are found in online supplemental appendix 2.

10.1136/bmjgh-2022-011104.supp1 Supplementary data

The search was conducted on 1 December 2022. Trial registries and data from unpublished articles were not included. Duplicated records were deleted first using the software (EndNote V.20.2) or manually if identified later. Additionally, a collaborative exchange was initiated with maternal and newborn health networks and international organisations via solicitations over email listings and social media to expand the search to unpublished studies and tools.

Three researchers (NM, RK and MR-C) conducted title and abstract screening, reviewed full-text articles and extracted data using a standardised form developed for the purpose of this study. For each step (title/abstract review, full-text review), two reviewers independently reviewed each paper. The extraction was done by one reviewer. Any discrepancies were discussed with a third party (AP) until consensus was reached. We extracted data on study design, data collection methods, study population, timing and care type, and data collection instruments and measure domains. For manuscripts published in a language other than English, a coauthor fluent in that language reviewed the manuscript. The study protocol was registered and published on protocol.io (ID: 50559; dx.doi.org/10.17504/protocols.io.bvk7n4zn).

### Data synthesis and mapping

Data were extracted using Covidence Extraction V.2.0 and exported to Stata V.17. 0 (StataCorp. 2021. Stata Statistical Software: Release V.17. College Station, Texas: StataCorp LLC). First, basic information of the article and methods was extracted. We grouped the manuscripts by the concepts measured, either experience of care (indirect or physiological) or satisfaction. We extracted information on all the tools, measures and topic guides that were used and characterised them according to the type of respondents, the period in the continuum of care, the data collection method and place (ie, facility or home), whether it reported validation, the number of items and any theoretical frameworks underpinning the instrument.

To identify the subcategories (or ‘domains’) of the concepts of experience of and satisfaction with care covered in the tools, we used the WHO Quality-of-Care framework and its related standards as an organising guide.^1^ This framework comprises eight domains of quality of care, each domain supported by one standard of care. We specifically focused on standards four through eight, which pertain to the experience of care as opposed to the clinical provision of care. We adapted the *WHO Standards for improving the quality of care for small and sick newborns in health facilities*^13^ to encompass all newborns regardless of their health status at birth. We chose to use these standards as a framework due to their direct relevance to newborn care. Each standard contains multiple quality statements that outline the necessary components for compliance.

We mapped each question of the available tools or measures into the corresponding quality statement of the experience of care-related standards (standards four to eight), which allowed us to identify gaps in the measurement tools. For example, if a tool asked whether the parents received accurate information from health workers, we mapped the question within Standard 4: Quality statement 4.2 (all newborns and their carers experience coordinated care, with clear, accurate information exchange among relevant health and social care professionals and other staff). We then calculated the frequency with which each quality statement was covered by the available tools. If a tool covered aspects that did not align to any of the quality statements or provided additional information to an existing quality statement, we presented them separately.

We did not assess quality or risk of bias for the included articles as the objective of this review was to scope the literature and describe the breadth of instruments and measures used to assess experience of and satisfaction with care and was not concerned with the magnitude or directionality of bias in any outcome variable.

### Patient and public involvement

Patient and public involvement was not a component of this project. As a scoping review, patients were not involved in this research, however findings will be discussed with stakeholder groups.

## Results

### Overall characteristics of included studies

**Figure 1:**
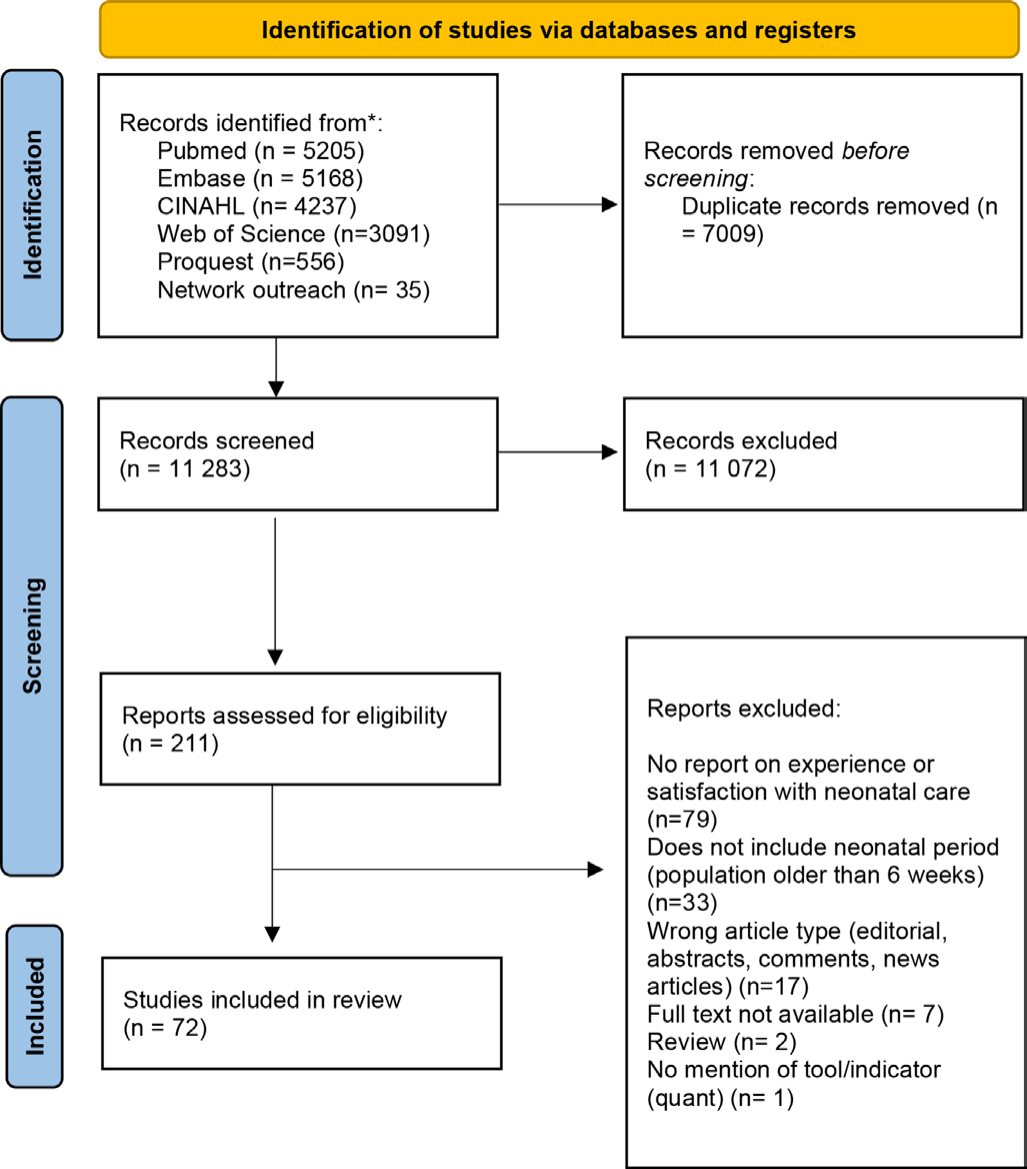
PRISMA flow diagram. PRISMA, Preferred Reporting Items for Systematic Reviews and Meta-Analyses.

We identified 18 257 records from the database searches, and an additional 35 through network outreach strategies (figure 1). Of these, 72 records met the eligibility criteria and were included in the synthesis. All studies were published between 2010 and 2022 and were mostly reported in English except three in Spanish.^14–16^

**Figure 2:**
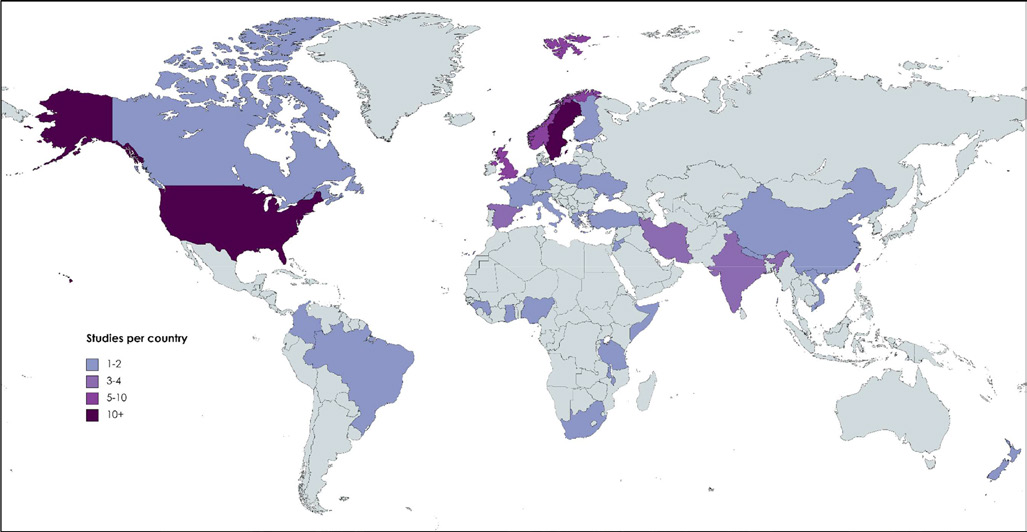
Distribution of included studies by country and region.

Table 1 reports the characteristics of included studies. In summary, the 72 included studies were conducted in 34 countries (figure 2). Most studies were conducted in high-income countries (51/72 studies), with 21 studies conducted in low-income or middle-income countries.

**Table 1:** Characteristics of the included studies

Study characteristics	Total (%)
Total number of included articles	72 (100)
Type of study	
Quantitative	48 (66.7)
Qualitative	17 (23.6)
Mixed methods	7 (9.7)
Main study aim*	
Instrument validation/testing	5 (6.9)
Measurement/ exploration	62 (86.1)
Evaluation (ie, of programme or policy)	7 (9.7)
Number of study participants (Median (min to max))	260 (11 to 4295)
Type of respondents*	
Mother	50 (69.4)
Parents	37 (51.4)
Other caregivers (not parents)	2 (2.7)
Health workers	6 (8.3)
Newborn	17 (23.6)
Newborn conditions at birth*	
Well baby	20 (27.8)
Low birth weight or preterm	39 (54.2)
Other birth comorbidities	9 (12.5)
Not reported	4 (5.6)
Timing in continuum of care	
Postnatal care (birth to discharge)	36 (50.0)
Postnatal care on day 3 (48–72 hour)	4 (5.6)
Postnatal care on day 7–14	2 (2.8)
Postnatal period (up to 6 weeks)	11 (15.3)
Other or not reported	19 (26.4)
Place of care	
Facility	63 (87.5)
Enroute to facility	1 (1.4)
Home	4 (5.6)
Not reported	4 (5.6)
Type of facility	
Primary or secondary level care	9 (12.5)
Tertiary level care	36 (50.0)
Not reported	27 (37.5)
Main concept measured*	
Indirect experience	36 (50.0)
Physiological experience	16 (22.2)
Satisfaction	23 (31.9)

*More than one could be present in the same study.

From the included studies, almost half (35/72) studied parents or carers’ experience with newborn care,^17–51^ although only 19 had this as a primary aim. Of those 19, eight focused on overall parental experience with newborn care,^22 27 30 32 38 42 43 45^ the remaining eleven focused on specific aspects of care, such as kangaroo mother care (KMC) provision,^37 41^ discharge education,^26^ postnatal diagnoses,^18 24^ end of life care^21 25 31^ or home-based postnatal care.^47 48 50^ Additionally, 16 studies measured newborn experience during hospital stay through physiological markers or measures of pain and comfort during routine procedures such as skin-to-skin contact, bathing, cobedding or heel prick, among others.^52–67^ Only two articles focused on mistreatment of the newborn during the immediate postpartum period, both using an adapted version of the Bohren *et al*^68^ typology of mistreatment (developed to measure maternal experience).^69 70^

Satisfaction with care was covered in 25 studies,^14–17 27 38 43 45 48 55 71–85^ but only 15 had it as primary aim,^14 15 38 43 45 55 72 74–77 79 81–83^ with seven focused on overall satisfaction and the remaining focused on the care received during stay in neonatal intensive care unit (NICU) or on other specific aspects such as rooming-in, infrastructure, education at discharge, the administration of routine interventions or home-based care.

Most of the studies covered the care between birth and discharge or birth to 72 hours,^14 18–20 23 26 27 30 33–35 41 43 46 49 51–58 61 62 64 67 69–73 76 78–80 82–85^ four studies reported on follow-up care at home,^16 47 48 50^ one on care during ground ambulance transport^37^ and five studies covered end-of-life care.^25 36 42 72 84^ We found no studies that reported on home birth.

### Tools and measures in included studies

Many of the included studies measured satisfaction or experience with the overall care received by the newborns, while others focused on specific aspects of care. These aspects included the type and quality of the information provided to the parents or carers by health workers or facility staff (n=8 articles),^14 15 24 26 27 36 43 46^ such as the receipt of education on infection prevention and control, explanation of the machines, monitors and alarms in NICU, counselling on how to express milk, information on diagnosis and management of neonatal conditions as well as predischarge counselling on breast feeding and care at home. Other aspects related to the comfort of newborns during routine practice and the use of adequate pain management strategies that are responsive to the newborns physiological and behavioural cues like skin-to-skin contact, non-nutritive sucking, facilitated tucking or twins cobedding (n=17 articles).^52–54 56–67 72 86^ The participation of parents or carers as decision-makers and active participants in newborn care interventions such as KMC was also considered (n=14 articles).^14 20 21 23 27 29 31 37 39–41 43 46 65^ The emotional support and attitudes of the health worker either towards the newborn or the family were analysed (n=11 articles),^14 15 19 21 24 27 34 43 46 51 76^ including end-of-life care and comprehensive approaches to palliative care (n=5 articles).^25 36 42 72 84^ Continuity of care during newborns’ stay in the healthcare facility, during transfer to another facility, or after discharge (n=7 articles)^16 28 30 37 47 48 50^ was also examined, as was the facility environment including access, physical space and lightning (n=1 article).^14^ Finally, 14 studies explored the experience or satisfaction with care in the context of family-integrated or family-centred care, including staff support, parental involvement in care, information sharing, discharge preparation and feeding support,^22 25 27 29 44 46 49 55^ respectful newborn care^69 70^ or overall quality of care.^24 38 82 85^

Additional details of included studies are found in online supplemental appendix 3, including type of study, study aim, aspects of care studies and concepts measured.

### Mapping of tools and measures by WHO Standards of Care

The studies included in the analysis used a total of 76 different tools and measures, of which almost 50% had undergone varying degrees of validation (n=34). Only 17 of the 72 studies provide a theoretical framework to justify their choice of domains within the tools.^17 19 22 25 28 30 32 33 37 44 46 55 64 69–72^ We extracted 53 individual tools or measures that were available and could be mapped against the WHO Standards of Care (table 2).^13^ These were either presented within the body of the article, as online supplemental materials, in the references of articles or requested to the authors. The available tools and measures were matched against the adapted standards 4 to 8 and their corresponding quality statements from the adapted WHO Standards.^13^ Table 3 shows the proportion of tools that covered each standard. The most commonly covered domains of care were those that related to effective communication, meaningful participation, responsive care to family’s needs and preferences and parental involvement, aligned with quality statements 4.1 to 4.3.

**Table 2:** Characteristics of tools by concept measured and explored

Tool/measures	Aspect of care studied	Respondents	Self-developed	Validated	Number of items	Available	Country	Reference
Indirect experience of care (quantitative tools)								
FiCare Questionnaire	Family integrated care	Parents	Yes	Yes	33	Yes	UK	Bradford-Duarte and Gbinigie^22^
Parental Stressor Scale-NICU (PSS-NICU)	Family centred care	Parents	No	Yes	22	Yes	Italy, Poland	De Bernardo *et al*^55^, Zych *et al*^51^
SMS questionnaire	Family centred care	Parents	Yes	NA	8	Yes	Finland, Sweden, Norway, Estonia, Spain, Italy	Raiskila *et al*^46^
Parent’s Perception of Continuity Scale (PPCS)	Continuity of care	Parents	Yes	Yes	27	Yes	USA	Epstein *et al*^28^
Structured questionnaire	Overall and end of life care	Parents	Yes	No	35	Yes	Germany	Bohnhorst *et al*^21^
CARE-Q instrument	Quality of nursing care	Parents	No	Yes	46	Yes	Colombia	Jaramillo-Santiago *et al*^34^
Listening to parents survey	Bereavement and end of life care	Mothers	No	Yes	84	Yes	UK	Redshaw and Henderson^42^
Mail questionnaire	Kangaroo mother care	Mothers/ Parents	Yes	No	24/NA	No	Sweden	Blomqvist and Nyqvist^20^, Calais *et al*^23^
Telephonic survey	Discharge education	Mothers	Yes	No	NA	No	Spain	Herrero-Morin *et al*^15^
Parent Survey Feedback	Bereavement care and follow-up support	Parents	Yes	No	NA	No	USA	Levick *et al*^36^
Structured questionnaire	Medical technology in NICU	Parents	Yes	Yes	65	No	Sweden	Lantz and Ottosson^35^
Labour observation tool	Newborn practices during immediate postnatal care	Observation	No	Yes	15	Yes	Ghana, Guinea, and Nigeria	Sacks *et al*^70^
EN-BIRTH respectful newborn care tool	Respectful newborn care	Mothers	No	Yes	13	Yes	Nepal	Gurung *et al*^69^
DigiFCC	Family centred care	Parents	No	Yes	9	Yes	Canada	Lebel *et al*^49^
Indirect experience of care (mixed-methods tools)								
Parent’s experience survey	Diagnoses information and support	Parents	Yes	NA	42	Yes	UK	Costa *et al*^24^
Topic guide	Overall neonatal care	Mothers	Yes	NA	NA	No	Tanzania	Mbwele *et al*^38^
Indirect experience of care (qualitative exploratory tools)								
Clinical Interview for Parents of High-Risk Infants (CLIP)	Parental involvement (facilitated tucking)	Parents	No	Yes	8	Yes	Finland	Axelin *et al*^19^
Family Interview Guide	Diagnoses information and treatment	Family	Yes	No	20	Yes	USA	Atwood *et al*^18^
Semi-structured interview guide	Postnatal discharge education	Mothers	Yes	Yes	7	Yes	Tanzania	Dol *et al*^26^
Interview guide	NICU hospitalisation, end of life and palliative care	Parents	Yes	No	11	Yes	USA	Currie^25^
Parental questionnaire	Bereavement care and support	Parent	Yes	Yes	35	Yes	France	Einaudi *et al*^31^
Parent Focus Group Interview Guide	Overall neonatal and home care	Parents	Yes	No	37	Yes	UK	Franck *et al*^30^
Interview guide	Interhospital transportation	Parents	Yes	No	11	Yes	Sweden	Lundqvist *et al*^37^
Interview guide	NICU admission	Parents	Yes	No	12	Yes	UK	Russell *et al*^43^
Interview guide	Overall care	Parents	Yes	No	10	Yes	UK	Sawyer *et al*^45^
15-minute tool	Communication	Mothers	Yes	NA	NA	No	South Africa	Horwood *et al*^32^
Interview guide	Kangaroo mother care	Parents, Mothers	Yes	NA	NA	No	Sweden, Malawi, Brazil	Noren *et al*^39^, Nyondo-Mipando *et al*^40^, Pereira Viana *et al*^41^
Interview guide	Father’s involvement	Fathers	Yes	NA	NA	No	Canada	Feeley *et al*^29^
Interview guide	Home-based postnatal care	Parents	Yes	No	6	Yes	Norway	Aune *et al*^47^
Interview guide	Home-based postnatal care	Fathers	Yes	NA	5	No	Sweden	Johansson *et al*^48^
Interview guide	Home-based postnatal care	Fathers	Yes	NA	NA	No	Norway	Solberg *et al*^50^
Physiological experience of care (quantitative tools/measures)								
Behavioral Indicators of Infant Pain (BIIP)	Pain management	Clinical assessment	No	Yes	3	Yes	Brazil, Turkey	Rodrigues and Guinsburg^63^, Caka and Gözen^53^
Neonatal Infant Pain Scale (NIPS)	Pain management	Clinical assessment	No	Yes	6	Yes	Brazil	Rodrigues and Guinsburg^63^
Premature Infant Pain Profile (PIPP)	Pain management	Clinical assessment	No	Yes	7	Yes	Brazil, USA, Germany, Taiwan	Rodrigues and Guinsburg^63^, Mitchell *et al*^86^, Russell *et al*^64^, Liaw *et al*^56^, Hsieh (2018)
Neonatal Facial Coding System (NFCS)	Pain management and comfort	Clinical assessment	No	Yes	10	Yes	Taiwan	Liaw *et al*^57^
Douleur Aiguë Nouveau-né (DAN) Scale	Pain management	Clinical assessment	No	Yes	3	Yes	China	Liu *et al*^58^
Neonatal Skin Condition Score	Pain management	Clinical assessment	No	Yes	4	Yes	Turkey	Caka and Gözen^53^
Newborn Comfort Behaviour Scale	Pain management and comfort	Clinical assessment	No	Yes	6	Yes	Turkey	Özdel and Sari^61^
Salivary cortisol, urinary oxytocin	Stress management	Clinical assessment	NA	NA	NA	Yes	Canada, Sweden, USA, Ukraine	Campbell-Yeo *et al*^54^, Mörelius *et al*^60^, Mitchell *et al*^86^, Pavlyshyn *et al*^67^
Physiological signals (heart rate (HR), respiratory rate (RR), oxygen saturation, body temperature)	Stress and pain management	Clinical assessment	NA	NA	NA	Yes	UK, Turkey, Taiwan, USA, Germany, Jordan	Boyle *et al*^52^, Caka and Gözen^53^, Liaw *et al*^56^, Liaw *et al*^57^, Lowey *et al*^59^, Ranger *et al*^62^, Shattnawi and Al-Ali^65^, Russell^64^
Crying duration	Stress and pain management	Clinical assessment	NA	NA	NA	Yes	Turkey, Taiwan, Jordan, USA	Caka and Gözen^53^, Liaw^57^, Shattnawi *et al*^65^
Sleep patterns	Stress and pain management	Clinical assessment	NA	NA	NA	Yes	USA, Jordan, UK, USA	Loewy *et al*^59^, Shattnawi and Al-Ali^65^, Boyle *et al*^52^, Russell *et al*^64^
Facial expression	Stress and pain management	Clinical assessment	NA	NA	NA	Yes	UK, Taiwan, USA	Boyle *et al*^52^, Liaw *et al*^56^, Russell *et al*^64^
Body posture and movements	Stress and pain management	Clinical assessment	NA	NA	NA	Yes	UK, Taiwan, USA	Boyle *et al*^52^, Liaw *et al*^56^, Lowey *et al*^59^, Russell *et al*^64^
Suckling patterns	Stress and pain management	Clinical assessment	NA	NA	NA	Yes	USA	Lowey *et al*^59^
Satisfaction with care (quantitative tools)								
Postnatal Interview Childbirth Satisfaction questionnaire	Essential newborn care in humanitarian settings	Parents	Yes (adapted)	Yes	20	Yes	Somalia	Amsalu *et al*^17^
Parent satisfaction questionnaire	Family centred care	Parents	Yes (adapted)	No	19	No	Iran	Bastani *et al*^71^
Bereaved Parent Satisfaction and Unmet Needs Questionnaire	End of life care	Parents	Yes	No	29	Yes	USA	Baughcum *et al*^72^
Pediatric Quality of Life Inventory (PedsQL) Healthcare Satisfaction Module 4.0	End of life care	Parents	No	Yes	24	Yes	USA	Baughcum *et al*^72^
Parent satisfaction survey	Overall care in NICU	Parents	Yes	No	24	Yes	Spain	Capdevila Cogul *et al*^14^
Satisfaction survey	Family centred care	Parents	No	Yes	9	Yes	Italy	De Bernardo *et al*^55^
Short assessment of patient satisfaction (SAPS)	Overall care in NICU	Parents	No	Yes	7	Yes	India	Dhingra^73^
Picker Institute NICU survey	Overall care in NICU	Parents	No	Yes	12	Yes	Greece	Galanis *et al*^74^
Neonatal Satisfaction Survey (NSS-13)	Overall care	Parents	Yes	Yes	13	Yes	Norway	Hagen *et al*^75^
Satisfaction survey	Maternal participation in NICU	Mother	Yes	No	26	No	USA	Holditch-Davis *et al*^76^
Neonatal Index of Parental Satisfaction (NIPS)	Overall care in NICU	Mothers	No	Yes	24	Yes	Iran	Kadivar *et al*^77^
Satisfaction questionnaire with the neonatal care services and hospital stay comfort	Rooming-in	Mothers	Yes	No	NA	No	Iran	Kazemian *et al*^78^
Care items	NICU practices	Parents/Health workers	Yes	No	92	No	The Netherlands	Latour *et al*^79^
NICU satisfaction survey	Telemedicine in NICU	Parents	Yes	No	10	Yes	USA	Makkar *et al*^80^
Parental satisfaction with quality of neonatal care	Overall care	Parents	Yes	No	22	Yes	Vietnam	Nguyen *et al*^81^
NICU satisfaction survey	NICU infrastructure	Parents	Yes	No	42	No	USA	Stevens *et al*^82^
Satisfaction questionnaire	Care at step-down ward	Mother	Yes	No	35	Yes	India	Taneja *et al*^83^
EMpowerment of PArents in THe Intensive CareNeonatology (EMPATHIC-N)	Information sharing and communication	Parents	Yes (adapted)	Yes	30	Yes	New Zealand	Williams^33^
Quality indicators and Parental Satisfaction with Palliative Care Instrument—Postnatal Scale	End of life and palliative care	Parents	Yes (adapted)	Yes	41	Yes	NA	Wool^84^
Satisfaction survey with home visit	Home-based postnatal care	Mothers	Yes	No	6	Yes	Spain	Feijoo-Iglesias *et al*^16^
Visual analogue scale	Home-based postnatal care	Fathers	NA	NA	NA	No	Sweden	Johansson *et al*^48^
Satisfaction with care (mixed methods tools)								
Parental dissatisfaction questionnaire	Overall care	Parents	Yes	Yes	60	No	Sweden	Ellberg *et al*^27^
Topic guide	Overall care	Mother	Yes	No	NA	No	Tanzania	Mbwele *et al*^38^
Parental survey	Information sharing	Parents	Yes	No	NA	Yes	New Zealand	Williams *et al*^33^
Satisfaction with care (qualitative tools)								
Interview guide	NICU admission	Parents	Yes	No	12	Yes	UK	Russell *et al*^43^
Interview guide	Overall care	Parents	Yes	No	10	Yes	UK	Sawyer *et al*^45^
Interview guide	Family-centred care	Family	Yes	No	24	Yes	India	Sarin and Matia^44^
Survey on satisfaction with EENC implementation	Early essential newborn care	Parents	Yes	No	NA	No	China	Wang *et al*^85^

CARE-Q, Caring Assessment Instrument; EENC, Early Essential Newborn Care; NA, not available or not known; NICU, Neonatal intensive care unit; SMS, Short Messaging Service.

**Table 3:** Mapping of available tools into the WHO standard and quality statement of care by concept measured

Standard	Quality statement		Experience of care		Satisfaction (n=19)
			Indirect (n=20)	Physiological (n=14)	
**Standard 4:** Communication with newborns and their families is effective, with meaningful participation, and responds to their needs and preferences, and parental involvement is encouraged and supported throughout the care pathway.	4.1	All carers of newborns are given information about the newborn’ s illness and care, so that they understand the condition and the necessary treatment.	70%	7%	79%
	4.2	All newborns and their carers experience coordinated care, with clear, accurate information exchange among relevant health and social care professionals and other staff	80%	7%	79%
	4.3	All carers are enabled to participate actively in the newborn’s care through family-centred care and kangaroo mother care, in decision-making, in exercising the right to informed consent and in making choices.	80%	7%	74%
	4.4	Carers of newborns and staff understand the importance of nurturing interaction with the newborn, recognise and respect newborn behaviour and cues and include them in care decisions	50%	0%	53%
	4.5	All carers receive appropriate counselling and health education about the current illness and transition to kangaroo mother care follow-up, community care and continuous care, including early intervention and developmental follow-up.	70%	7%	42%
	4.6	In humanitarian and fragile settings, including outbreak and pandemic situations, special consideration is given to the specific psychosocial and practical needs of newborns and their carers	0%	0%	5%
**Standard 5**: Newborns’ rights are respected, protected and fulfilled, without discrimination, with preservation of dignity at all times and in all settings during care, transport and follow-up	5.1	All newborns have equitable access to healthcare services, with no discrimination of any kind.	10%	0%	5%
	5.2	The carers of all newborns are made aware of and given information about the newborn’s rights to health and healthcare	40%	7%	79%
	5.3	All newborns and their carers are treated with respect and dignity, and their right to privacy and confidentiality is respected	45%	7%	37%
	5.4	All newborns are protected from any physical or mental violence, injury, abuse, neglect or any other form of maltreatment.	15%	93%	32%
	5.5	All newborns have their birth registered and have an identity	0%	7%	0%
	5.6	All newborns who die and all stillbirths have their deaths registered	0%	0%	16%
**Standard 6**: All newborns are given developmentally supportive care and follow-up, and their families receive emotional and psychosocial support that is sensitive to their needs and strengthens their capability	6.1	All newborns stay with their carers, with minimal separation, and the role of carers is recognised and supported at all times during care, including rooming-in during hospitalisation.	35%	7%	42%
	6.2	All newborns born preterm or with a low birth weight receive kangaroo mother care as soon as possible after birth, and the parents are supported in its provision	35%	0%	16%
	6.3	All newborns receive appropriate developmental supportive care, and their families are recognised as partners in care	30%	0%	58%
	6.4	All families receive care in an environment in which their socioeconomic, emotional, and cultural needs are respected and supported	50%	7%	53%
	6.5	All newborns receive appropriate, coordinated developmental follow-up with minimal disruption to family life and routines	40%	7%	26%
**Standard 7:** For every newborn, competent, motivated, empathetic, multidisciplinary staff are consistently available to provide routine care, manage complications and provide developmental and psychological support throughout the care pathway.	7.1	All newborns have access to a sufficient multidisciplinary workforce, including health professionals, allied health, and support staff, at all times according to standard levels of care.	20%	7%	21%
	7.2	Health professionals, allied health and support staff have appropriate skills to support the health, psychological, developmental, communication and cultural needs of newborn and their families	50%	7%	68%
	7.3	All staff working in neonatal units of the health facility have the necessary knowledge, skills and attitudes to provide infection prevention and control, basic resuscitation, kangaroo mother care, safe feeding and medications and positive interaction with newborns and communication with carers	20%	7%	42%
**Standard 8:** The health facility has an appropriate physical environment, with adequate water, sanitation, waste management, energy supply, medicines, medical supplies and equipment for routine care and management of complications in newborns	8.1	Newborns are cared for in a safe, secure, well-maintained, organised physical environment that is appropriately designed to provide kangaroo mother care and family-centred care according to standard levels of care	25%	14%	47%
	8.2	Water, sanitation, hand hygiene and waste disposal facilities are easily accessible, functional, reliable, safe, and sufficient to ensure strict infection control and meet the needs of newborns, carers and staff	0%	0%	26%
	8.3	Equipment designed specifically for the medical care and developmental and emotional support of newborns is available at all times	0%	0%	32%
	8.4	Adequate stocks of medicines and medical supplies specific for newborns are available for routine care and for management of complications	0%	0%	11%
	8.5	All carers of newborns have access to a dedicated area with supportive elements including adequate space for kangaroo mother care, family-centred care, privacy for mothers to express breast milk and facilities for hygiene, cooking and laundry	25%	0%	37%

Green boxes indicate that the quality statement is covered in 50% or more of available tools. Yellow boxes indicate that the quality statement is covered in 25% to <50% of available tools. Red boxes indicate that the quality statement is covered by less than 25% of available tools.

More than half of the tools that addressed satisfaction with care measured the provision of developmentally supportive care, including minimal separation or rooming-in, in an environment that met the family’s socioeconomic, emotional and cultural needs (standard 6).

However, there were few or no tools that measured the standards related to newborns’ rights and preservation of dignity (standard 5), availability of staff for adequate care provision and developmental and psychological support (standard 7) and adequate physical environment (standard 8).

### Additional aspects covered by tools and measures

The mapping exercise also highlighted aspects of newborns’ experience of and satisfaction with care included in the measurement tool that expands on the WHO standards and their respective quality statements (box 1). Twenty-one studies measured newborn pain and stress as measures of newborn experience relating it both to adequate pain management during routine practices—including non-nutritive suckling, breast feeding or music during heel prick — and stress-free environmental conditions such as optimal sound and light levels in the NICU.

Box 1 **Salient aspects measured in tools related to experience of and satisfaction with care emerging from the mapping exercise**

**Newborn comfort and pain management:**

Measures of pain during routine care, including adequate stress-free environment with optimal sound and light levels.

**Information and communication:**

Measures of adequate, comprehensive and comprehensible information on the newborn condition and care received by parents and carers, including education on infection prevention and control; explanation of the machines, monitors and alarms in the NICU; counselling on how to express milk; predischarge counselling on breast feeding and information on diagnosis and management of neonatal conditions.

**Parental participation:**

Measures of parental participation and decision-making in the care of their newborns, including in the provision of interventions such as kangaroo mother care.

**Continuity of care:**

Measures of continuity of care including consistency of information provided to the carers, and consistency of interactions between carers and health workers.

Measures of adequate receipt of information and support on breastfeeding and care after discharge by carers to feel prepared to continue care at home.

Measure of participation of carers in the decision of transferring the newborn, with information on the place of transfer, facilitating any logistical needs and allowing the provision of kangaroo mother care during transport.

**Emotional support to all carers**

Measures of compassionate care and management of parental and carers’ stress and anxiety about newborns conditions and hospitalisation, including offer of time off during hospitalisation.

Measures of equal treatment to all carers, ensuring a private and supportive environment to stay by the newborn.

**End of life care**

Measures of compassionate palliative care provision, sensitive to the family’s culture and traditions, with bereavement information and support, keepsake boxes, etc.

Additionally, the type and quality of predischarge information for the parents were collected in the tools used in 18 studies as an aspect of satisfaction with care, with a particular emphasis on how to care for the newborn in the home after discharge tailored to newborn’s health conditions, and on supportive routine counselling on breastfeeding.

Eighteen studies focused on continuity of care and adequate referral as another important aspect encompassing experience and satisfaction with care. This included the continuity within the facility, ensuring consistent information across health providers, ongoing relationship between service users and providers or enabling transfer in KMC position and the referral to other facilities allowing parental involvement in the decision of transferring the newborn and the considerations of logistical needs for the family.

Other aspects that emerged less frequently in the findings were the inclusion of family visits as an integral part of neonatal care experience (n=4), involving a private and supportive environment to visit the newborn in NICU as well as the incorporation of compassionate and sensitive end of life care (n=3) according to the family’s culture and traditions, including adequate bereavement information and support, keepsake boxes or other elements that impact family’s experience.

## Discussion

This review aimed to identify available tools that measure experience of or satisfaction with neonatal care, to give a comprehensive landscape of the current state of measures and instruments used in research. We identified a total of 72 articles discussing experience and/or satisfaction with different aspects of newborn care, including indirect and physiological experience. However, substantial variation existed in how experience and satisfaction were conceptualised and operationalised across studies. Our review identified two aspects that should be considered when developing tools to measure the experience of newborn care. First, non-verbal and physiological cues provide a means for newborns to express their own experience and should not be overlooked, despite their inability to communicate verbally. Second, the link between a newborn’s experience of care and that of their mother or carer should be taken into account to gain a more comprehensive understanding of the care received by the newborn. While these aspects can be beyond the scope of our review, they could be fundamental factors to consider when developing tools to measure the experience of newborn care.

An innovative finding of this review is the inclusion of non-verbal cues, such as behavioural responses, physiological signals and pain profiles as markers of newborn experience. Despite the challenges posed by newborns being unable to communicate their needs and experiences verbally, attention to the infant’s pain indicators, sleep patterns, sounds and skin condition can be objectively measured to assess the quality of care received. Creating a comforting and compassionate environment, with adequate levels of sound and lighting, pain management, minimising unnecessary interventions and promoting skin-to-skin contact to reduce parent–newborn physical separation appear as critical aspects of family-centred, nurturing care and essential components of the newborn experience. However, additional discussion is necessary to achieve an appropriate balance between the value of physiological cues as markers of newborn experience and the feasibility of including them in assessment tools. While non-verbal cues can provide valuable information about the quality of care received, health workers may lack the resources, training or time needed to consistently and accurately conduct these assessments. The subjectivity of the person conducting the assessment and the frequency of assessments are also important considerations. Additionally, the invasiveness of some measures, such as cortisol levels, may not be appropriate for routine use. Therefore, it is essential to carefully consider the feasibility, reliability and invasiveness of non-verbal cues before incorporating them into assessment tools. Ultimately, striking a balance between the frequency of assessments and their practicality is vital, and care providers must receive proper training to ensure accurate and consistent assessments of these cues.

When mapping the tools against the WHO domains, domains related to parental participation and decision-making in care, including effective and supportive communication and counselling from health workers (Standard 4), and related to the role of health workers and parents in ensuring continuity of care and receiving coordinated care (Standard 6) were prevalent across studies related to both experience and satisfaction. Active parental involvement in caring for their infants during hospital stay and at home was identified as an important item to measure, as it can influence not only the care received by the newborn but also parent–infant attachment and parent’s psychological stress. These findings are supported by the recently published scoping review by Ndwiga and collaborators that shows that experience of care in young children up to 24 months is highly driven by positive communication with hospital staff, nurturing care and parental engagement in the care of the newborn.^87^

We found a large number of tools available for measuring experience and satisfaction with newborn care, including 69 individual tools and seven categories of physiological and behavioural measures. However, our analysis revealed significant variation in the way these tools are used. Only 17 of the 72 studies provide a theoretical framework to justify their choice of domains.^17 19 22 25 28 30 32 33 37 44 46 55 64 69–72^ This lack of theoretical underpinning may be due to a recent paradigm shift from focusing solely on provision of care to incorporating experience as essential components of family-centred, high-quality care.^88^ Additionally, the period and place of newborn care covered by the studies varied greatly including birth to discharge, the immediate postnatal period or different periods within the first 2 weeks during hospital stay, either during NICU stay or until death. Follow-up care, care in the community or in lower level facilities, was neglected by the included studies. These variations in measurement hinder comparability between studies and highlight the lack of standardised processes for data collection, analysis and reporting of experience of care across the continuum of postnatal care. We recognise that not all measures and tools can be standardised across different types of care, and that certain domains may be more specific to a particular type of experience, such as care at discharge, care in the NICU or end-of-life care. However, certain core domains of experience are cross-cutting, such as communication, emotional support, pain management and respect for values and preferences. Standardising measures used to assess these core domains could enhance the comparability of experience of care data across different types of care.

While our review does not address all aspects of standardisation, we acknowledge that these may be important factors to consider in future research. This review also identified a shortage of validated instruments to measure overall experience of or satisfaction with newborn care, with less than half of the included papers using validated tools. However, the limited availability of the tools in the public domain or the difficult access to them can hinder the validation of these tools in different countries and contexts. Lack of validation can pose a challenge to cross-study comparability, exposing the results to subjectivity and unreliability. Addressing subjectivity when it comes to experience with neonatal care becomes crucial when designing these tools. If not adequately addressed, the instrument may not measure the aspect of care intended, but rather the influence of adverse neonatal conditions or outcomes on carers’ overall experience or satisfaction.^9^ Therefore, a future agenda should include steps to identify globally-agreed standardised measures of the experience of care, and the testing of some of the prioritised measures that can be used across different populations of newborns and across the newborn period, regardless of whether they are term and healthy or sick and small and to contribute towards improving the measurement of experience of care and monitoring quality and programmes.

This study is not without limitations. There were numerous challenges encountered in compiling tools in a standardised manner. First, the categorisation of tools within a specific domain of care was difficult as certain tools lacked clear definitions of what they intended to measure and whether it fell within the realm of experience of care. In many studies, particularly qualitative, experience and satisfaction were used interchangeably, so we relied on Larson *et al’s*^9^ definition to make the final decision of the concept being measured. Another challenge arose from the significant overlap between provision and experience when it comes to newborn care. Interventions such as skin to skin contact or breastfeeding are an indispensable part of the provision of essential newborn care, but they also contribute to the overall experience of both the carers and the newborns. We included tools that measured interventions involving parents and carers’ participation as they may play a significant role on carer’s overall experience. However, we recognise that other interventions may also impact parents’ and carer’s experiences. Finally, our aim was to understand experience from both the newborn and the carers, and, thus, health workers were intentionally excluded from the study population as they may be influenced by clinical judgement or health systems constraints. However, we recognise that their input will be essential in designing interventions to improve experience of care. Despite our systematic search and efforts to reach out to networks to retrieve all available literature, and not applying any language exclusion criteria, it is possible that some studies were missed, particularly those not in English. Finally, we did not assess the quality of each included study; future research will be needed to identify methodological shortcomings and biases present in available tools.

This scoping review provides a comprehensive compilation of both validated and non-validated measures and instruments of experience and satisfaction with newborn care. This can serve as an important basis for the prospective work of developing clear, consistent and comprehensive tools and measures, and integrated tools that capture mother/parents/carers perspectives as well as tools that capture newborns’ experiences. To that end, we explored and identified pertinent domains of care that can be put forward for further research on experience and satisfaction with newborn care, to ensure that a clear theoretical framework is created. We did this assessing literature from all regions, from different periods in the continuum of care, and including newborns with different health conditions.

The concepts of experience and satisfaction with newborn care still hold substantial variation both conceptually and operationally. Although the WHO standards offer a good direction to identify gaps, there is a clear need to create a unified and comprehensive understanding and typology on what constitutes a positive experience of newborn care. Comprehensive and validated instruments measuring all aspects of care are still needed. Developing a robust theoretical ground will be fundamental to the design and selection of standardised tools and measures that inform policy and programme implementation. With the launch of the updated WHO postnatal care guidelines^11^ and guidelines on the care of preterm and low-birth-weight infants,^89^ there is a new opportunity to enable national and global programme monitoring and implement appropriate action for improving postnatal care uptake and experience for both mother and newborns and end preventable maternal and newborn mortality.

## Data Availability

All data relevant to the study are included in the article or uploaded as supplementary information.
