# Supplementary material for: Measuring experience of and satisfaction with newborn care: a scoping review of tools and measures
Source: BMJ Glob Health. 2023 May 9;8(Suppl 2):e011104. doi: 10.1136/bmjgh-2022-011104 (PMC10186411; doi:10.1136/bmjgh-2022-011104)
Supplement: Supplementary data [file bmjgh-2022-011104supp001.pdf]

Appendix 1. PRISMA-ScR checklist

SECTION	ITEM	PRISMA-ScR CHECKLIST ITEM	REPORTED ON PAGE #
**TITLE**			
Title	1	Identify the report as a scoping review.	Page 1
**ABSTRACT**			
Structured summary	2	Provide a structured summary that includes (as applicable): background, objectives, eligibility criteria, sources of evidence, charting methods, results, and conclusions that relate to the review questions and objectives.	Page 1
**INTRODUCTION**			
Rationale	3	Describe the rationale for the review in the context of what is already known. Explain why the review questions/objectives lend themselves to a scoping review approach.	Page 2
Objectives	4	Provide an explicit statement of the questions and objectives being addressed with reference to their key elements (e.g., population or participants, concepts, and context) or other relevant key elements used to conceptualize the review questions and/or objectives.	Page 3
**METHODS**			
Protocol and registration	5	Indicate whether a review protocol exists; state if and where it can be accessed (e.g., a Web address); and if available, provide registration information, including the registration number.	Page 4
Eligibility criteria	6	Specify characteristics of the sources of evidence used as eligibility criteria (e.g., years considered, language, and publication status), and provide a rationale.	Page 3
Information sources*	7	Describe all information sources in the search (e.g., databases with dates of coverage and contact with authors to identify additional sources), as well as the date the most recent search was executed.	Page 3
Search	8	Present the full electronic search strategy for at least 1 database, including any limits used, such that it could be repeated.	Appendix 2
Selection of sources of evidence†	9	State the process for selecting sources of evidence (i.e., screening and eligibility) included in the scoping review.	Page 3-4
Data charting process‡	10	Describe the methods of charting data from the included sources of evidence (e.g., calibrated forms or forms that have been tested by the team before their use, and whether data charting was done independently or in duplicate) and any processes for obtaining and confirming data from investigators.	Page 4-5
Data items	11	List and define all variables for which data were sought and any assumptions and simplifications made.	Page 3
Critical appraisal of individual sources of evidence§	12	If done, provide a rationale for conducting a critical appraisal of included sources of evidence; describe the methods used and how this information was used in any data synthesis (if appropriate).	Page 5
Synthesis of results	13	Describe the methods of handling and summarizing the data that were charted.	Page 3-4
**RESULTS**			

SECTION	ITEM	PRISMA-ScR CHECKLIST ITEM	REPORTED ON PAGE #
Selection of sources of evidence	14	Give numbers of sources of evidence screened, assessed for eligibility, and included in the review, with reasons for exclusions at each stage, ideally using a flow diagram.	Page 5-6, Fig 1
Characteristics of sources of evidence	15	For each source of evidence, present characteristics for which data were charted and provide the citations.	Page 6-7
Critical appraisal within sources of evidence	16	If done, present data on critical appraisal of included sources of evidence (see item 12).	n/a
Results of individual sources of evidence	17	For each included source of evidence, present the relevant data that were charted that relate to the review questions and objectives.	Page 6-7
Synthesis of results	18	Summarize and/or present the charting results as they relate to the review questions and objectives.	Page 7-10
**DISCUSSION**			
Summary of evidence	19	Summarize the main results (including an overview of concepts, themes, and types of evidence available), link to the review questions and objectives, and consider the relevance to key groups.	Page 12-13
Limitations	20	Discuss the limitations of the scoping review process.	Page 12-13
Conclusions	21	Provide a general interpretation of the results with respect to the review questions and objectives, as well as potential implications and/or next steps.	Page 13
**FUNDING**			
Funding	22	Describe sources of funding for the included sources of evidence, as well as sources of funding for the scoping review. Describe the role of the funders of the scoping review.	Page 14

Appendix 2. Pubmed Search Strategy

The following search strategy for Pubmed was adapted for the other search engines:
(((((((("Infant Health"[Mesh]) OR "Postnatal Care"[Mesh] OR "Newborn Care"[Mesh] OR "Newborn Health"[Mesh] OR "Neonatal Care"[Mesh] OR "Neonatal Health"[Mesh]) OR "Maternal-Child Health Services"[Mesh]) OR "Infant Care"[Mesh]) OR "Child Health Services"[Mesh]))))) AND ((((((experience[tiab] OR experiences[tiab] OR patient-centered[tiab] OR “woman centered”[tiab] OR “women centered”[tiab] OR “client centered”[tiab] OR satisfaction[tiab] OR “social support*”[tiab] OR “emotional support*”[tiab] OR “provider choice”[tiab] OR “choice of provider”[tiab] OR “wait time*”[tiab] OR affordability[tiab] OR dignity[tiab] OR respect[tiab] OR privacy[tiab] OR confidentiality[tiab] OR discrimination[tiab] OR communication[tiab] OR disrespect[tiab] OR abuse[tiab] OR mistreatment[tiab] OR perception*[tiab] OR "positive experience"[tiab]))))

Filter: publication date after 1st January 2010

Appendix 3. Additional characteristics of included studies

	Author	Year	Country	Respondents/ Participants	Type of study	Study aim	Aspect of care studied	Newborn conditions at birth	Concept measured	Tool/Measure	Tool availabl e	WHO Standards covered in study (if tool/tools available)
1	Amsalu, R.	2019	Somalia	Parents	Quantitative	To investigate the feasibility and effectiveness of the Field Guide in improving newborn-care practices and quality of care at the primary health facility level in humanitarian settings	Essential newborn care in humanitarian settings	All newborns	Satisfaction	Postnatal Interview Childbirth Satisfaction questionnaire	Yes	4,5,6,7,Other*
2	Atwood, A.C.	2016	USA	Family	Qualitative	To better understand the family experience during the newborn hospitalization for Neonatal Abstinence Syndrome (NAS) through a qualitative approach.	Neonatal abstinence syndrome treatment	Babies with Neonatal Abstinence Syndrome	Satisfaction	Family Interview Guide	Yes	4,5,6,7,8,Other *
3	Aune, I	2021	Norway	Parents	Qualitative	To examine parents' experiences of early discharge and home visits by the postnatal ward midwife, in cases where the mother and baby have been discharged within 24 h after birth.	Home visit after early discharge	Healthy babies	Satisfaction	Interview guide	Yes	4,6,Other*
4	Axelin, A.	2010	Finland	Parents	Qualitative	To describe and understand how mothers utilize the opportunity to actively participate in their preterm infants' pain care with Facilitated Tucking by Parents (FTP)	Parental involvement (Facilitated tucking by parents)	Preterm babies	Satisfaction	Clinical Interview for Parents of High-Risk Infants (CLIP)	Yes	4,5,6,7,Other*
5	Bastani	2015	Iran	Parents	Quantitative	To determine the effect of family-centred care including maternal participation, presence, and information about neonatal care on maternal satisfaction and neonatal readmission.	Family centered care on readmission	Preterm babies	Satisfaction	Parent satisfaction questionnaire	No	
6	Baughcum	2020	USA	Parents	Mixed-methods	To examine parent perceptions of their infant's EOL experience (eg, symptom burden and suffering) and satisfaction with care in the NICU	End of life care	Infant death	Satisfaction	Bereaved Parent Satisfaction and unmet needs questionnaire/ pEDSqL	Yes	4,5,6,7,

7	Blomqvist	2010	Sweden	Mothers	Quantitative	To characterise the first infants treated with continuous KMC from birth to discharge, to investigate aspects of the practical application of KMC and to explore the mother's experiences during the implementation period.	Kangaroo Mother Care	Moderately preterm and ill newborn infants	Satisfaction	Mail questionnaire	No	
8	Bohnhorst B.	2015	Germany	Parents	Mixed-methods	To investigate the impact of prenatal counseling on subsequent parents' experiences during in-patient care of their infant(s) and whether feelings of parents with deceased infants are different in principle	Overall experience with extremely premature babies and EOL care	Very preterm babies	Satisfaction	Structured questionnaire	Yes	4,5,6,Other*
9	Boyle, E. M.	2017	UK	Parent and staff	Quantitative	to determine (i) difficulty perceived by staff and parents inassessing comfort/persistent pain in babies, (ii) strategies employed when no clinical toolis used and (iii) variation between clinicians' assessments.	Pain and comfort of newborn	Babies admitted to NICU	Physiological experience	Parent questionnaire	Partially	5,8,Other*
10	Bradford-Duarte	2020	UK	Parents and health worker	Mixed-methods	To gain feedback from previous parents in order to identify barriers and facilitators to a positive parental experience, areas for improvement and to consider specific changes in practice to implement.	Family Integrated Care	Babies admitted to NICU	Indirect experience	FiCare Questionnaire	Yes	4,6,7,8,Other*
11	Caka, S. Y.	2017	Turkey	Newborns	Quantitative	To determine the effect of traditional tub bathing (TTB) and swaddled bathing (SB) methods on physiological measurements and Newborn Infant Pain Scale (NIPS) score of healthy newborns born at 38+ weeks.	Swaddled and traditional tub bathing methods	Healthy term newborns	Physiological experience	Neonatal Infant Pain Scale (NIPS)	Yes	5,Other*
12	Calais, E.	2010	Sweden, Norway	Mother/Father	Quantitative	To explore factors that promote or hinder Skin to Skin Contact (SSC) between parents and their healthy full term infants during the first days after birth.	Kangaroo Mother Care	Healthy full term infants	Indirect experience	Mail questionnaire	No	
13	Campbell-Yeo M.	2010	Canada	Newborn	Quantitative	To determine the effect of co-bedding on preterm twins stress response during a tissue breaking procedure (heel lance) in the NICU.	Co-bedding for stress reduction	Stable preterm twin babies admitted to NICU	Physiological experience	Salivary cortisol	Yes	5,Other*
14	Capdevila Cogul E.	2012	Spain	Parents	Quantitative	To evaluate the degree of satisfaction of parents, and to value if there is any relation between the severity of	NICU care	Babies admitted to NICU	Satisfaction	Parent satisfaction survey	Yes	4,5,6,7,8,Other*

						newborn health and parent's satisfaction						
15	Cardoso Rodrigues	2012	Brazil	Newborn	Quantitative	To assess whether a routine painless procedure becomes perceived as painful by premature infants with 28 to 32 weeks of gestational age during their first 28 days of postnatal life while hospitalized in an intensive care unit.	Pain management	Preterm babies admitted to NICU	Physiological experience, Satisfaction	Behavioral Indicators of Infant Pain (BIIP)/Neonatal Infant Pain Scale (NIPS)	Yes	5,Other*
16	Costa, B.	2019	UK	Parents	Quantitative	To examine the experiences of a large sample of parents in relation to their child's postnatal diagnosis and neonatal Cleft Lip/Palate care.	Diagnoses and care of cleft lip/palate	Newborns with cleft lip/palate	Satisfaction	Parent's experience survey	Yes	4,5,7,Other*
17	Currie, E. R.	2015	USA	Parents	Qualitative	To explore and describe parent experiences related to their infant's NICU hospitalization, EOL care, and PPC consultation.	NICU hospitalization, EOL care and palliative care	Infant death	Satisfaction	Interview guide	Yes	Other*
18	De Bernardo, G.	2017	Italy	Parents	Quantitative	To compare satisfaction and stress levels between parents in an Family-Centred Care group and a non Family-Centred Care group.	Family centered care in NICU	Babies admitted to NICU	Indirect experience, Satisfaction	Satisfaction survey	Yes	4,5,6,7,8,Other *
19	Dhingra, P.	2020	India	Parents	Quantitative	To study the concerns of parents whose babies were admitted to NICU, parental satisfaction level about the services provided and their understanding and knowledge at discharge	NICU care	Babies admitted to NICU	Satisfaction	Short assessment of patient satisfaction (SAPS)	Yes	4,5,6,7,
20	Dol, A.	2019	Tanzania	Mothers and urse midwives	Qualitative	To explore the experience of newborn care discharge education at a national hospital in Dar es Salaam, Tanzania from the perspective of mothers and nurse midwives.	Postnatal discharge education	All babies	Satisfaction	Semi-structured interview guide	Yes	4,Other*
21	Einaudi, M.A.	2010	France	Parents	Qualitative	To understand the parental response to perinatal death by describing the experiences of the families involved.	Bereavement care	Infant death	Satisfaction	Parental questionnaire	Yes	4,5,6,7,Other*
22	Ellberg, L	2010	Sweden	Parents	Mixed-method	To describe how new parents experience postpartum care.	Overall postnatal care	Term babies	Satisfaction	Parental dissatisfaction questionnaire	No	

23	Epstein, E.G	2013	USA	Parents	Quantitative	To develop and test a scale of parental perceptions of nursing continuity of care in the newborn intensive care setting and to characterize the association between parents; perceptions and chronological nursing continuity.	Continuity of care in NICU	Babies admitted to NICU	Indirect experience	Parent's perception of continuity Scale (PPCS)	Yes	4,5,6,7,Other*
24	Feeley	2012	Canada	Fathers	Qualitative	To explore what fathers perceive to be facilitators or barriers to their involvement with their infants.	Father's involvement	Babies admitted to NICU	Indirect experience	Interview guide	No	
25	Feijoo-Iglesias, M.B.	2021	Spain	Mothers	Quantitative	To detect whether the strategy of early and voluntary dischage and home visit by midwife during the Covid-19 pandemic had an adverse effect on women and newborns	Home visit after early discharge	Term babies	Satisfaction	Satisfaction survey with home visit	Partially	4,6
26	Franck, L. S.	2017	UK	Parents	Qualitative	To discover parents' views, experiences, concerns, and recommendations about the care provided to them and their babies throughout the perinatal and neonatal healthcare journey in a UK context.	Overall perinatal, neonatal and home care	Peterm babies	Indirect experience	Parent Focus Group Interview Guide	Yes	4,6,Other*
27	Galanis, P.	2016	Greece	Parents	Quantitative	To evaluate parents' satisfaction with care provided in NICU, explore factors associated with parent's satisfaction with care and identify specific unmet needs of parents with infants in NICU	Overall NICU care	Babies admitted to NICU	Satisfaction	Picker Institute NICU survey	Yes	4,5,7,Other*
28	Gurung, R.	2021	Nepal	Mothers	Quantitative	To analyse exist survey reported experience of care after hospital birth (selected maternal respectful care components) as part of EN-BIRTH multicountry validation study	Respectful care/ Mistreatment	All babies	Mistreatment	EN-BIRTH questionnaire	Yes	4,5,6,7,8,Other *
29	Hagen, I.	2014	Norway	Parents	Quantitative	To develop and validate a survey to investigate parents' satisfaction with neonatal wards in a population of parents of children with a gestation age of 24 weeks to 3 months after full-term birth.	Overall satisfaction with care	Preterm babies admitted to NICU	Satisfaction	Neonatal Satisfaction Survey – NSS-13	Yes	4,5,6,7,8,Other *
30	Herrero-Morin, J.	2015	Spain	Mothers	Quantitative	To determine parents satisfaction with the information received at discharge, identify information that the parents would have liked to receive and determine what factors affect the demand for information	Discharge education	All babies	Indirect experience	Telephonic survey	No	

31	Holditch-Davis, D.	2013	USA	Mothers	Quantitative	To examine mothers' satisfaction with administering interventions for their preterm infants and with the helpfulness of the study nurse by comparing the ATVV intervention (massage with auditory, tactile, visual, and vestibular stimulation), kangaroo care, and education about the equipment needed at home.	Maternal participation in NICU	Preterm babies	Satisfaction	Satisfaction survey	No	
32	Horwood	2019	South Africa	Mothers	Qualitative	To explore mothers and health workers experiences of care provided in neonatal units in district hospitals in order to identify opportunities to improve Quality of Care.	Communication with health providers	Babies admitted to NICU	Indirect experience	15-minute tool'	No	
33	Hsieh K	2018	Taiwan	Newborn	Quantitative	To investigate if instilling breast milk (BM) or dextrose water into oral cavity can reduce the procedural pain of heel stick in preterm neonates.	Pain management	Preterm babies	Physiological experience	Premature infant pain profile (PIPP)	Yes	5
34	Jaramillo Santiago, L. X.	2018	Colombia	Parents	Quantitative	To identify the perceptions of parents of neonates hospitalized regarding the quality of nursing care	Quality of Nursing care in NICU	Babies admitted to NICU	Indirect experience	CARE-Q instrument	Yes	4,5,6,7,
35	Johansson M.	2022	Sweden	Fathers	Mixed-methods	To explore and describe fathers' experiences of a newly implemented Swedish home-based post-natal care model	Home-based postnatal care model	Healthy babies	Indirect experience, Satisfaction	Interview guide/ Visual Analogue Scale	No	
36	Kadivar, M.	2016	Iran	Mothers	Quantitative	To evaluate the effect of narrative writing on the satisfaction of the mothers with care in the neonatal intensive care unit (NICU) during their neonates' hospitalization.	NICU hospitalization	Babies admitted to NICU	Satisfaction	Neonatal index of Parental Satisfaction (NIPS)	Yes	4,5,6,7,Other*
37	Kazemian, M.	2016	Iran	Mothers	Quantitative	To investigate the effects of rooming-in on icteric neonates regarding the length of hospital stay, maternal satisfaction level, and complications of hospital stay.	Rooming-in	Icteric newborns	Satisfaction	Satisfaction questionnaire with the neonatal care services and hospital stay comfort	No	
38	Lantz, B.	2012	Sweden	Parents	Quantitative	To examine to what extent the different medical technological products commonly used in the NICU are perceived by parents as being obstacles to their wish to interact with their babies	Medical technology in NICU	Babies admitted to NICU	Indirect experience	Structured questionnaire	No	

39	Latour, J.M.	2010	The netherlands	Parents/Health workers	Quantitative	To identify NICU nurses and physicians perceptions of parental satisfaction with care issues and to reach a consensus on the identified issues.	NICU practices	NA	Satisfaction	Care items	No	
40	Lebel V.	2022	Canada	Parents	Quantitative	To determine if parental presence, involvement in infant care, holding, skin-to-skin contact (SSC), perceived family-centered care, depression symptoms, and sociodemographic characteristics are associated with the emotional closeness of parents with an infant hospitalized at the NICU.	Family centered-care	Preterm babies admitted to NICU	Indirect experience	DigiFCC	Yes	4,5,7,Other*
41	Levick , J .	2017	USA	Parents	Quantitative	To describe a comprehensive approach to delivering bereavement services to NICU families, as well as education and support to NICU staff.	Bereavement care and follow up support	Infant death	Indirect experience	Parent Survey Feedback on bereavement program	No	
42	Liaw J	2010	Taiwan	Newborn	Quantitative	To compare the efficacy of three strategies on newborns's pain, physiological parameters, and cry duration, before, during, and after their first IM injection of hepatitis B vaccine.	Non-nutritive suckling for preterm pain	Preterm babies	Physiological experience	Premature Infant Pain Profile (PIPP)	Yes	5,Other*
43	Liaw, J.	2011	Taiwan	Newborn	Quantitative	To examine the effectiveness of non-nutritive sucking on preterm infant pain, changes in infant behaviour and frequency of abnormal physiological signals during heel stick procedures in Taiwan.	Non-nutritive suckling for preterm pain	Preterm baby	Physiological experience	Neonatal Facial Coding System (NFCS)	Yes	5,Other*
44	Liu, M.	2013	China	Newborn	Quantitative	To investigate the effect of skin contact between mother and child in pain relief of full-term newborns during heel blood collection.	Pain management (KMC for pain relief)	Ful term newborn	Physiological experience	Douleur Aiguë Nouveau-né (DAN) Scale	Yes	5,Other*
45	Loewy, J.	2013	USA	Newborn	Quantitative	To study how live elements of music such as rhythm, breath, and parent-preferred lullabies may affect physiologic function in premature infants.	Stress and live music	Preterm babies	Physiological experience	HR, RR, SpO2, Sleep pattern, Activity level, Suckling pattern	Yes	Other*
46	Lundqvist, P.	2021	Sweden	Parents	Qualitative	To illuminate parents' experiences of holding their infantin a kangaroo position during neonatal ground ambulance transport	Interhospital transport (in KMC)	NA	Indirect experience	Interview guide	Yes	4,6,8,Other*

47	Makkar A.	2020	USA	Parents	Quantitative	To evaluate the safety and efficacy of premature infant treatment managed by hybrid telemedicine versus conventional care.	Use of telemedicine in NICU	Preterm babies	Satisfaction	NICU satisfaction survey	Yes	4,5,6,8,
48	Mbwele, B.	2013	Tanzania	Mothers	Mixed-methods	To assess mothers experiences, perception and satisfaction of neonatal care in the hospitals of the Kilimanjaro region of Tanzania.	Overall neonatal care	All babies	Indirect experience, Satisfaction	Topic guide	No	
49	Mitchell, A.J	2013	USA	Newborn	Quantitative	1. To determine whether stress in preterm infants, measured with salivary cortisol,decreases after five days of Kangaroo Care (KC) compared to five days of Standard Care (SC). 2.To determine whether kangaroo care provides sustainable pain relief beyond the period of skin-to-skin holding.	Management of pain and stress (kmc)	Preterm babies	Physiological experience	Salivary cortisol	Yes	5
50	Mörelius, E.	2015	Sweden	Newborn	Quantitative	To evaluate the effect of almost continuous SSC for late preterm infants (32-35 weeks gestation) on stress levels	KMC	Late preterm infants	Physiological experience	Salivary cortisol	Yes	5,Other*
51	Nguyen, A.T.B	2020	Vietnam	Parents	Quantitative	To assess and compare parental satisfaction and its determinants in two purposely chosen facilities: the provincial hospital of Thanh Hoa province, and the national hospital in Hanoi Capital.	Overall neonatal care	Preterm babies	Satisfaction	Structured questionnaire: Parental satisfaction with quality of neonatal care	Yes	4,5,7,8,Other*
52	Noren J.	2018	Sweden	Mothers	Qualitative	To describe Swedish mothers' experiences of KMC	KMC	Preterm babies	Indirect experience	Interview guide	No	
53	Nyondo-Mipando, A. L.	2020	Malawi	Caregivers	Qualitative	To explore the experiences of caregivers in the implementation of KMC.	KMC	Stable newborns	Indirect experience	Topic guide	No	
54	Özdel D	2018	Turkey	Newborn	Quantitative	To determine the effects of the KC and prone positions in preterm infants during feeding onthe residual volume, vital signs and comfort	Newborn comfort	Preterm babies	Physiological experience	Newborn Comfort Behavior Scale	Yes	5,7,8,Other*
55	Pavlyshyn, H.	2022	Ukraine	Newborn	Quantitative	To investigate how skin-to-skin contact (SSC) can influence the biologic stress levels in preterm infants in the NICU by assessing cortisol and oxytocin levels.	Newborn stress	Preterm babues	Physiological experience	Salivary cortison, Urinary oxytocin	Yes	5
56	Pereira Viana, M.R.	2018	Brazil	Mothers	Qualitative	To describe the experience of mothers of premature infants in the Kangaroo Mother Method and to analyze the experience of mothers of premature	KMC	Preterm babies	Indirect experience	Interview guide	No	

						infants in the Kangaroo Mother Method						
57	Ranger A.	2018	Germany	Newborn	Quantitative	To examine the effects of pentatonic live music on the cardiac and respiratory physiology of preterm neonates and on the level of anxiety of their mothers.	Stress and music	Preterm babies	Physiological experience	Desaturations, HR and RR	Yes	5,Other*
58	Redshaw M.	2018	UK	Mothers	Quantitative	To better understand parent's perceptions and experiences of care when the baby dies, and how parents's views and experiences might differ in relation to the gestational age at which their baby was born	Bereavement and EOL care	Preterm babies	Indirect experience	Listening to Parents survey	Yes	4,5,6,7,8,Other *
59	Russell G.	2014	UK	Parents	Qualitative	To explore parents' experiences and satisfaction with care during and immediately after the birth of their very premature baby	NICU admission	Preterm babies	Indirect experience, Satisfaction	Interview guide	Yes	5,Other*
60	Russell K.,	2015	USA	Newborn	Quantitative	To investigate the effects on premature and early term infants receiving neuroprotective care using a weighted maternally-scented parental simulation device	Neuroprotective care(using a weighted maternally-scented parental simulation device)-physiological stability	Preterm babies	Physiological experience	Premature Infant Pain Profile (PIPP)	Yes	4,5,Other*
61	Sacks E	2021	Ghana, Guinea, and Nigeria	Newborn	Quantitative	To describe the care received by neonates up to 2 h after birth in health facilities across three countries in west Africa using a standardised observation tool and to identify factors associated with these practices	Mistreatment	All babies	Mistreatment	Labour observation tool	Yes	5,6,Other*
62	Sarin, E	2019	Delhi	Family	Qualitative	To get a better understanding of the acceptability of family-centered newborn care from providers' and clients' perspectives, explore the integration of providers' and clients' activities in the neonatal unit, examine continuing care competencies of parents after discharge	Family centered newborn care model	Sick babies admitted to NICU	Indirect experience	Interview guide	Yes	4,6,Other*

63	Sawyer A	2012	UK	Parents	Qualitative	To explore parents' experiences and satisfaction with their care during very preterm birth, and to identify the domains associated with positive and negative experiences of care.	Care of very preterm baby	Very preterm babies	Indirect experience, Satisfaction	Interview guide	Yes	4,5,6,
64	SCENE research group	2016	Europe	Parents	Quantitative	To describe the current perceptions regarding quality of FCC from the perspectives of mothers, fathers and nurses perspectives in 11 European NICUs.	Family Centred Care in NICU	Preterm babies	Indirect experience	Text message questions	Yes	4,6,7,Other*
65	Shattnawi, K.K.	2019	Jordan	Newborn	Quantitative	To assess the effect of providing short periods and durations of SSC on premature infants' physiological and behavioural outcomes.	Pain management (KMC)	Preterm babies	Physiological experience	Weight gain, occurance of aneic eiposed, crying and sleeping patterns	Yes	5,Other*
66	Solberg, B.	2021	Norway	Fathers	Qualitative	Too explore fathers' experiences with the New Family home visiting program, to better understand their perspectives.	Home-based postnatal care model	All babies	Indirect experience	Interview guide	No	
67	Stevens, D	2011	USA	Parents	Quantitative	To test the hypothesis that parental satisfaction with neonatal intensive care is greater in a single-family room facility as compared with a conventional open-bay neonatal intensive care unit (NICU)	Infrastructure of NICU	Babies admitted to NICU	Satisfaction	NICU satisfaction survey	No	
68	Taneja, R	2020	India	Mother	Quantitative	To assess the satisfaction of mothers of neonates admitted in the neonatal stepdown ward of a public sector hospital	Care at step down ward	Preterm low birth weight babies	Satisfaction	Satisfaction questionnaire	Yes	4,5,6,7,8,Other *
69	Wang, C	2022	China	Parents and stakeholders	Mixed-methods	To comprehensively explore the positive impacts of Early Essential Newborn care implementation.	Early Essential Newborn Care	All babies	Satisfaction	Qualitative survey on satisfaction of EENC implementation	No	
70	Williams, L.	2020	New Zealand	Parents	Quantitative	To evaluate parents perception of information sharing in neonatal care, to determine parents' preferred medium for health information and to identify priority content for inclusion in a smartphone application.	Information sharing	Babies admitted to NICU	Satisfaction	EMpowerment of PArents in THe Intensive CareNeonatolog y (EMPATHIC-N)	Yes	4,5,6,7,8,Other *

71	Wool, C.	2017	NA	Parents	Quantitative	To examine quality indicators and how they influence satisfaction with care as parents faced their infant's shortened life span.	Perinatal palliative care	Babies with life limiting conditions	Satisfaction	The Voice of Parents including Postnatal scale	Yes	4,5,6,7,8,Other*
72	Zych, B	2021	Poland	Parents	Quantitative	To assess the degree of parental stress and coping strategies in parents giving KMC to their babies hospitalized in NICU compared to the control group of parents not giving KMC	KMC during NICU admission	Babies admitted to NICU	Indirect experience	Parental Stressor Scale: Neonatal Intensive Care Unit (PSS-NICU)	Yes	4,5,6,7,8,Other*

**Other* refers to those tools that cover additional aspects of care not included in the WHO standards
